# A highly stable lyophilized mRNA vaccine for Herpes Zoster provides potent cellular and humoral responses

**DOI:** 10.1038/s41541-025-01093-1

**Published:** 2025-03-14

**Authors:** Raquel Munoz-Moreno, Viola Allaj, Eddie Gadee, Julie M. Button, Fernando Diaz, Mohan S. Maddur, Wei Chen, Cheng Hui Hu, Lyndsey Martinez, Andreas Giannakou, Adam Lee Campbell, Yana Miteva, Pengbo Guo, Bridget Huang, Shuai Shi, Jason Lotvin, Kristin Tompkins, Pirada Suphaphiphat Allen, Alicia Solórzano

**Affiliations:** Vaccine Research and Development, Pfizer, Pfizer, Pearl River, NY USA

**Keywords:** Immunology, Microbiology, Diseases, Medical research, RNA vaccines

## Abstract

Herpes zoster (HZ) is a painful vesicular rash that occurs upon varicella-zoster virus (VZV) reactivation in older adults and immunocompromised individuals. Although there is currently an approved vaccine for the prevention of shingles, its administration is commonly associated with high reactogenicity. This highlights the need to develop new vaccine alternatives with long lasting immunity and improved tolerability upon administration. In the present study, 10 different vaccine candidate designs using two different codon optimizations targeting the VZV glycoprotein E (gE) were generated. A subset of mRNA constructs were formulated into lipid nanoparticles and assessed for their ability to induce specific cellular and humoral immune responses following vaccination in mice. Notably, the selected mRNA vaccine candidates induced high levels of antibodies and robust CD4^+^ but also CD8^+^ immune responses. Moreover, we showed that our alternate lyophilized vaccine provides comparable immunogenicity to current liquid frozen formulations and is stable under long-term storage conditions.

## Introduction

Herpes zoster (HZ), or shingles, is an acute, localized, and painful vesicular rash that occurs as a result of varicella-zoster virus (VZV) reactivation^1^. Primary VZV infection typically occurs in children and causes varicella (chickenpox), which is characterized by vesicular skin lesions containing high concentrations of virions. Upon primary infection, VZV preferentially infects memory T cells, establishing latency in sensory ganglia and causing HZ upon reactivation mainly in older adults and immunocompromised individuals^2,3^. It has been estimated that the individual lifetime risk of developing HZ is about 30% in the general population in the US^4^, and its occurrence is generally attributed to an age-related decline in T cell-mediated immunity, putting older adults at a greater risk of severe disease and long-term sequelae^5^.

Complications associated with HZ are frequent and can significantly affect a patient’s quality of life and ability to perform daily activities. While VZV reactivation is usually restricted to a single dermatome, about one in ten adults with HZ will develop postherpetic neuralgia (PHN), a neuropathic pain syndrome that involves severe pain that can persist for several months to years following HZ rash resolution^4^. In the United States (US), HZ is responsible for around 96 deaths annually with most cases reported in older adults or patients with immunocompromising conditions^6^. Nationwide, HZ and its associated complications translate into $2.4 billion in productivity losses and direct medical costs annually for an unvaccinated population^7^. Globally, the incidence of HZ is on the rise, leading to billions of dollars in annual costs for the healthcare system and society due to lost productivity^8^.

Shingrix® (GlaxoSmithKline, Rockville, MD, US) is currently the only licensed vaccine on the US market for the prevention of HZ. It is a VZV gE protein-based subunit vaccine adjuvanted with AS01_B_ (a combination of liposomes and two immunostimulants)^9^. Shingrix® is administered intramuscularly in a two-dose regimen, with vaccine efficacy over 97% in reducing the risk of HZ in individuals 50 years of age or older^10,11^. While Shingrix® has shown very high efficacy, reactogenicity upon administration has been reported in 84.4% of participants 50 years of age or older who received the vaccine^10^. Frequent local reactions included pain, redness and swelling at the injection site, while the most common systemic reaction reported was myalgia, followed by fatigue, headache, fever, and gastrointestinal symptoms. Most symptoms were of mild-to-moderate intensity; however, in 17% of the recipients, the severity of the symptoms was reported to affect normal daily activities and could last up to three days^12^. In addition, the polysaccharide mixture QS21, a component of the AS01_B_ adjuvant system that is extracted from the bark of *Quillaja saponaria*, could potentially cause problems in the distribution supply chain since it is a limited resource that can only be found in the temperate regions of South America^13,14^. These factors imply there is a need to develop additional, easy-to-manufacture vaccines that cause fewer adverse reactions.

The unprecedented success of mRNA vaccines to prevent severe disease caused by severe acute respiratory syndrome coronavirus 2 (SARS-CoV-2), demonstrating high efficacy with an acceptable safety profile and scalable manufacturing^15,16^, set the groundwork for developing similar vaccines against other infectious diseases, including HZ. VZV modRNA vaccine candidates have been evaluated in animal models in several studies. For example, a VZV modRNA vaccine encoding a 573 amino acid (aa) C-terminal truncated VZV gE protein showed comparable immunogenicity to an adjuvanted protein vaccine in non-human primates (NHP)^17^. The C-terminal cytosolic tail included a Y569A mutation to modulate subcellular trafficking and expression of the VZV gE protein on the cell surface and in the Golgi^18^. A similar VZV gE mRNA vaccine tested in mice using different routes of immunization (intramuscular and subcutaneous) also demonstrated similar immunogenicity to an adjuvanted protein vaccine^19^. Additional studies have shown that an mRNA vaccine based on a full-length sequence-optimized gE was able to induce higher immunogenicity with a better safety profile when compared to Shingrix® in mice and rhesus macaques^20^. Similarly, Bhattacharya et al. recently reported that the immunogenicity and T cell responses elicited by three different mRNA vaccine candidates targeting VZV gE were also higher than Shingrix® in a mouse model^21^.

The wild-type (WT) gE protein (623 aa) accumulates in the trans-Golgi network (TGN) upon VZV infection^22^. gE is composed of four main domains: a signal peptide (SP) located at the N-terminal end, followed by an ectodomain, a transmembrane domain (TM) and finally a cytoplasmic tail (CT) at the C-terminal end. The CT of the gE protein contains three different motifs that have been implicated in its localization: 1) an AYRV sequence (aa 568 to 571) is required for targeting gE to the TGN; 2) a YAGL sequence (aa 582 to 585), resembling other YXXL endocytosis motifs, mediates gE internalization^23^ and 3) an “acid patch”, a cluster of acidic amino acids, including two serines (S593 and S595) and two threonines (T596 and T598), that have been shown to be involved in gE trafficking^18^.

We hypothesized that the localization of the gE WT protein in the TGN could be suboptimal to elicit a strong immune response. Therefore, we constructed a panel of modRNA vaccine candidates encoding different gE antigen designs aimed at manipulating the subcellular localization of the protein and evaluated their expression in vitro. Selected modRNA candidates were formulated in lipid nanoparticles (LNPs) and assessed for immunogenicity in preclinical mouse models to identify lead candidates for vaccine development. Additionally, a lyophilized formulation was evaluated as a convenient and viable clinical alternative to a frozen presentation.

## Results

### Rationale and generation of VZV gE modRNA constructs

The WT VZV gE is a type I integral membrane protein that is localized at the plasma membrane as well as internally, where it accumulates mostly in the Golgi, which could be a suboptimal localization for inducing robust immunity^24^. Therefore, we engineered alternative mutants to maximize expression levels of gE at the cell surface as well as secreted versions.

**Fig. 1:**
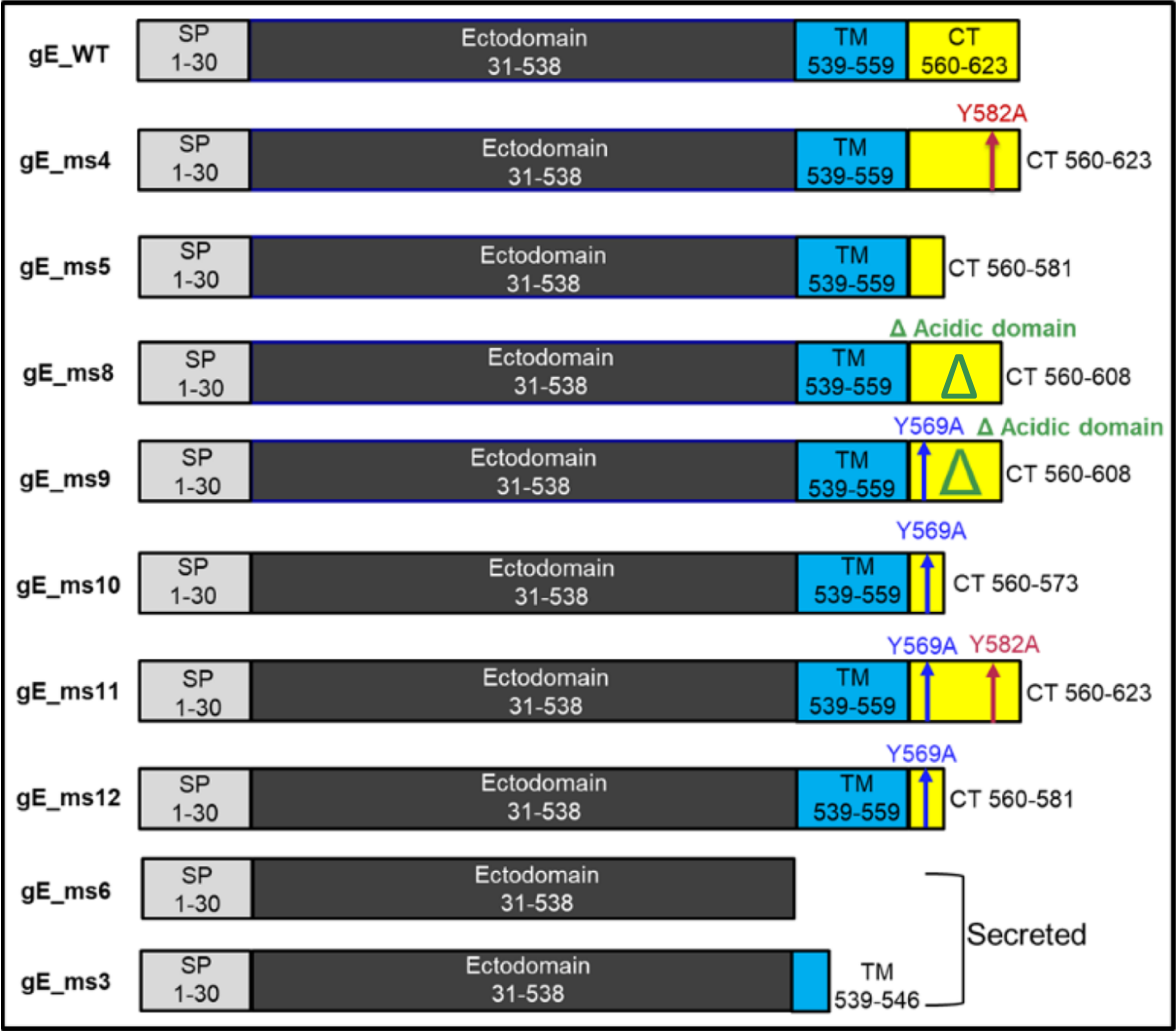
Design of mRNA constructs expressing different versions of varicella zoster virus (VZV) glycoprotein E (gE). Schematic representation of the different gE designs that were screened and evaluated in vitro. The different gE constructs are characterized for containing an N-terminal signal peptide (SP) followed by an ectodomain and a transmembrane domain (TM) that is present in all membrane anchored constructs (gE WT, ms4, ms5, ms8, ms9, ms10, ms11 and ms12) but is partially present or completely absent in the gE secreted versions (ms3 and ms6). Additionally, some of the designs contain a cytoplasmic tail (CT) with the Y569A mutation which is responsible for targeting the gE to the TGN (ms9, ms10, ms11 and ms12) and/or a deletion of a phosphorylated acidic amino acid cluster that is associated with Golgi localization (ms8 and ms9). Other CT truncations of different lengths were included in gE_ms5, ms10 and ms12 as illustrated in the diagrams.

A total of 10 different designs using two different codon optimizations were generated (Fig. 1). Some constructs such as the gE_ms4 and gE_ms11 variants are endocytic recycling mutants that contain the Y582A mutation. The tyrosine located at this position is part of a conserved YXXL motif which has been shown to be key for endocytosis and efficient internalization of the protein, so constructs lacking this residue are expected to express the gE mostly at the cell surface^23^. VZV gE_ms9, gE_ms10, gE_ms11 and gE_ms12 constructs include the Y569A mutation which is responsible for targeting gE to the TGN^25^. gE_ms8 and gE_ms9 designs contain a deletion of a phosphorylated acidic aa cluster in the CT domain associated with Golgi localization^22^. Additional CT truncations of different lengths were included in the gE_ms5, gE_ms10 and gE_ms12 vaccine construct designs. Secreted versions containing either a shortened TM (gE_ms3) or entirely lacking the TM domain (gE_ms6) were also included in the screening.

To maximize protein expression levels, two different codon optimizations were used. They are referred to as codon optimization 1 (Co1) or codon optimization 2 (Co2), where Co2 contains a higher GC content (~ 66%) when compared to Co1 (~58%). Both codon optimizations were tested in all the designs except for gE_ms8 which was exclusively Co1. All VZV modRNA vaccine candidates were generated by in vitro transcription (IVT) and full analytics including RNA integrity as well as capping percentage were assessed for each construct with results meeting target specifications.

### In vitro screening and selection of VZV gE vaccine candidates

**Fig. 2:**
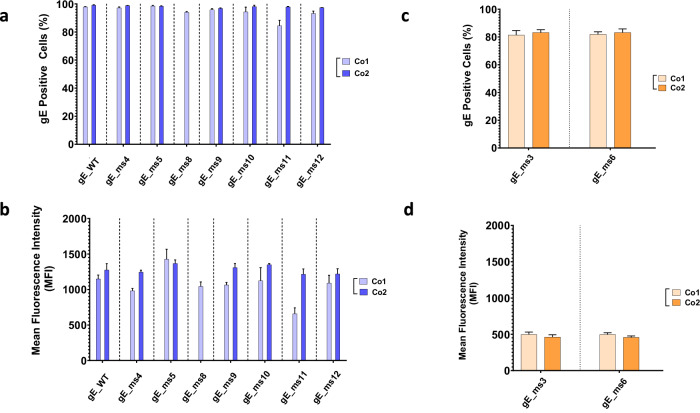
mRNA gE constructs in vitro screening and candidate selection. mRNA vaccine candidates generated were tested in HeLa cells. A total of 50 ng of RNA were transfected and analyzed 24 h later through indirect immunofluorescence assay (IFA). Cells were fixed, permeabilized and percentage positive cells and mean fluorescence intensity (MFI) were measured using a specific antibody against the VZV gE protein. **a** Percentage of gE positive cells and (**b**) mean fluorescence intensity (MFI) of membrane anchored mRNA VZV gE constructs were measured for two different codon optimized sequences based on different GC content (Co1 and Co2). **c** Percentage of gE positive cells and (**d**) MFI of soluble mRNA VZV gE constructs was measured for two different codon optimized sequences based on different GC content (Co1 and Co2).

Initial screening of the different VZV modRNA designs targeting the gE protein was performed in vitro in Vero cells using unformulated modRNA, also known as drug substance (DS). Further candidate selection was based on protein expression levels and subcellular localization patterns. The percentage of positive cells as well as protein expression levels determined by mean fluorescent intensity (MFI) were measured following RNA transfection. At the 50 ng dose, while almost 100% of the cells were gE positive across all constructs tested (Fig. 2a), different levels of protein expression (MFI) were observed for constructs containing modifications in the CT (Fig. 2b). In general, constructs with higher GC content (Co2) showed higher gE expression levels compared to their Co1 counterparts except for ms5 where Co1 trended slightly higher compared to the Co2 codon optimization.

Secreted candidates, gE_ms3 and gE_ms6, showed around 80% cells positive for gE at the 50 ng dose (Fig. 2c). However, total intracellular protein expression levels were significantly lower, as expected for modRNAs expressing secreted proteins, with no significant differences observed between the two codon optimizations tested (Fig. 2d).

**Fig. 3:**
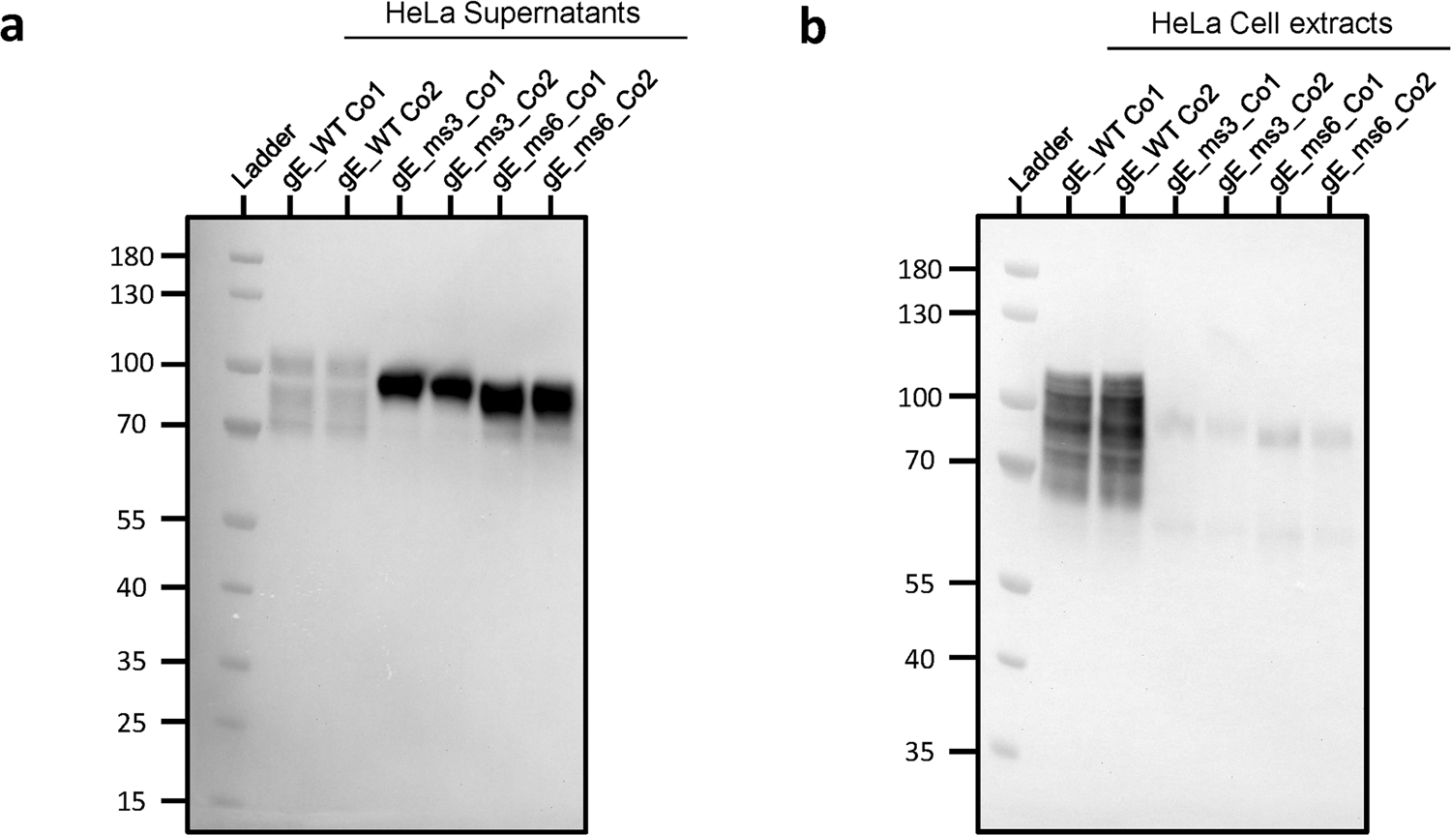
mRNA gE constructs expressing secreted versions of gE are highly expressed in cell supernatants. Western blot of cell supernatants (**a**) and cell extracts (**b**) after transfecting HeLa cells with 2.5 µg of RNA with the indicated gE-expressing mRNA constructs (gE WT, gE_ms3 and gE_ms6) that were synthetized with two different codon optimizations (Co1 and Co2). Samples were collected and analyzed 24 h after transfection. Both gE soluble versions (gE_ms3 and gE_ms6) were barely detected in total cell lysates but were instead detected at high levels in cell supernatants as opposed to gE WT version which was mostly detected in whole cell extracts.

To assess whether the low protein expression levels observed intracellularly were due to the gE protein being secreted, HeLa cells were transfected with the secreted candidates. This cell line derives from human cervical cancer cells and is known to be highly efficient for gene transfection^26^. HeLa cell supernatants as well as cell lysates were individually harvested 24 h post transfection and gE expression levels were analyzed by Western Blot (WB) (Fig. 3). Results showed that gE expression for gE WT Co1 and Co2 controls were mainly found in total cell lysates and in very low amounts in cell supernatants. Conversely, gE protein for gE_ms3 and gE_ms6 was barely detectable in total cell lysates and highly abundant in cell supernatants for both codon optimizations tested, thus demonstrating that gE proteins from modRNA constructs that partially or completely lack the TM domain can be efficiently secreted outside transfected cells. When comparing the amounts of secreted gE in the cell supernatant fraction, the signal for gE_ms6 was overall greater than the signal for gE_ms3 (that still retains part of the TM domain), suggesting that the gE_ms6 version was expressed to higher levels and/or was secreted more abundantly compared to gE_ms3 (Fig. 3a). Regarding both codon optimizations tested, gE_ms3 Co1 showed stronger signal than gE_ms3 Co2, while for gE_ms6, the two different codon optimizations showed comparable expression levels in the supernatants and were in both cases higher compared to the ms3 counterparts (Fig. 3a).

**Fig. 4:**
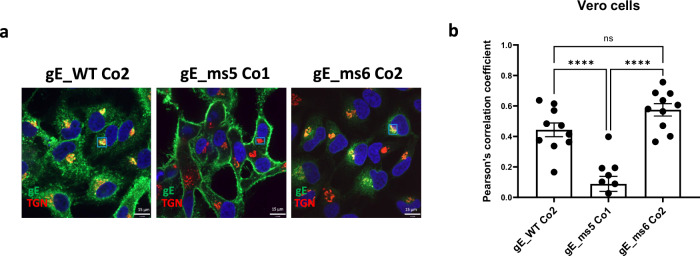
Subcellular localization and characterization of selected mRNA VZV gE vaccine candidates in Vero cells. VZV gE protein presence in the Golgi was assessed 24 h after transfection in Vero cells using spinning disk confocal microscopy. **a** DS from the three lead candidates: gE WT_Co2, gE_ms5 Co1 and gE_ms6 Co2 were transfected into Vero cells. 24 h later cells were washed, permeabilized and fixed. gE protein was detected using a human mAb against VZV gE and A488 secondary antibody. Golgi apparatus was stained using a specific antibody against N-acetylgalactosaminyltransferase 7 (GALNT7) and using A555 as a secondary antibody for detection. Nuclei were stained using DAPI (blue). Small rectangles represent the areas that were selected for analysis of colocalization. **b** A total of ten images were acquired per condition and colocalization levels between VZV gE protein and Golgi were measured for each mRNA construct using Pearson’s correlation coefficient based on regions of interest (ROI). One-way Analysis of Variance (ANOVA) with Tukey’s multiple comparison test was performed to determine statistical significance between the sample groups. ****p < 0.0001; ns: not significant.

Taking into account the total percentage of gE-positive cells, protein expression levels and the subcellular localization of the gE variants tested, three lead candidates were selected for further characterization using confocal microscopy: gE_WT Co2, gE_ms5 Co1 and gE_ms6 Co2 (Fig. 4a). Drug substance (DS) derived from these three candidates were transfected into Vero cells, a cell line widely used for imaging purposes, and co-stained for gE and polypeptide N-acetylgalactosaminyltransferase 7 (GALNT7), a protein that specifically accumulates at the Golgi apparatus. Results showed that VZV gE_WT Co2 was mainly expressed intracellularly and potentially associated with the endoplasmic reticulum (ER) as well as in the Golgi apparatus and cell membrane; gE_ms5 Co1 was highly expressed at the cell surface and gE_ms6 Co2 was poorly expressed inside the cells which is consistent with the secreted nature of this mutant (Fig. 4a). Additionally, we quantified gE colocalization levels between gE and the Golgi based on the Pearson’s correlation coefficient in Vero cells (Fig. 4b). Results showed low correlation values between gE and the Golgi apparatus for gE_ms5 Co1, since the VZV antigen was found mainly at the cell surface. Also, in agreement with the acquired immunofluorescence images, values were significantly increased in the gE WT version and were similarly high in the secreted mutant ms6 compared to gE_ms5 Co1, which is consistent with gE trafficking through the Golgi network before being secreted outside the cells thus showing low overall gE intracellular levels.

### In vitro expression of selected LNP-formulated VZV gE modRNA candidates

**Fig. 5:**
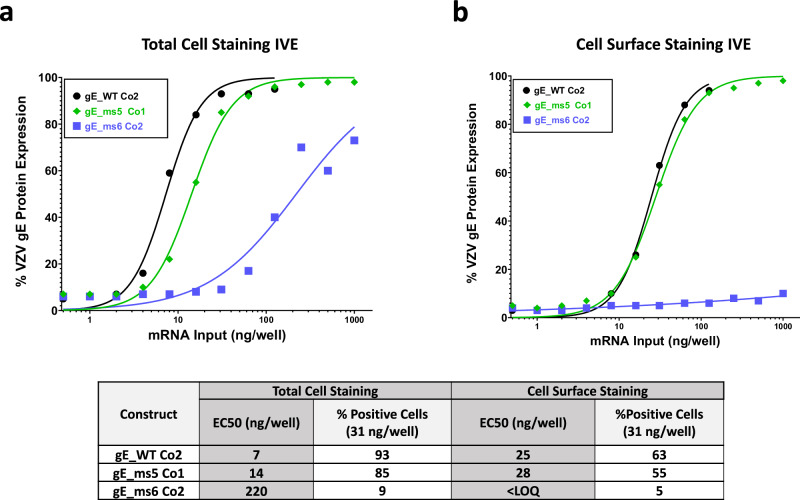
Total and cell surface in vitro expression assay (IVE) of selected mRNA VZV gE vaccine candidates in HEK 293T cells. In vitro expression of mRNA VZV LNP formulated material for the three proposed vaccines candidates gE WT_Co2, ms5_Co1 and ms6_Co2 was measured by flow cytometry in HEK 293T cells and whole gE expression levels (**a**) or gE levels in the cell surface (**b**) were detected using a specific antibody against VZV gE. EC50 values (ng/well) were calculated from each curve and are shown in a table together with the (%) positive cells at 31 ng dose for both conditions tested.

In vitro expression (IVE) of the top three VZV gE modRNA candidates was assessed to measure the potency of each selected construct once they were formulated into LNPs. HEK-293T cells were administered with the 3 different LNPs and were either fixed and permeabilized or just fixed to compare total gE intracellular levels versus surface protein levels through flow cytometry. The half maximal effective concentration (EC50) values, determined by total cell staining, showed very high potency levels for gE_WT Co2, followed closely by gE_ms5 Co1 whereas significantly lower values were observed for secreted version gE_ms6 Co2 (Fig. 5a). For cell surface staining, no differences were observed between the gE WT and ms5 versions while, as expected, gE protein was barely detected on the cell surface in the case of the secreted modRNA construct (Fig. 5b). These results are consistent with the previous confocal microscopy data and highlight the high correlation that can be found between unformulated and LNP-formulated modRNA material when it comes to modRNA performance.

### gE modRNA candidates elicited robust IgG levels and T cell responses in vivo

**Fig. 6:**
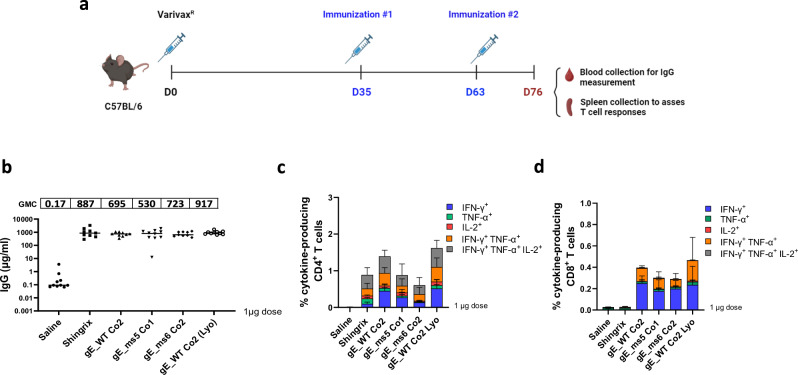
Immunogenicity and CMI responses elicited by mRNA VZV gE vaccine candidates in vivo*.* Female C57BL/6 mice were immunized with a full human dose of live attenuated VZV vaccine (Varivax®) to create preexisting immunity against VZV. Animals were subsequently immunized intramuscularly (IM) at day 35 and day 63 with 1 µg of our mRNA vaccine lead candidate LNP formulated material: gE WT_Co2 (frozen and lyophilized (Lyo) version), as well as frozen ms5_Co1 and ms6_Co2. Shingrix was also included and used as a positive control in the study. **a** Schematics of immunization and vaccination regimen schedule. Created in BioRender. Munoz-Moreno, R. (2024) https://BioRender.com/x92w358. **b** Blood was collected via submandibular route and gE-specific IgG antibodies (μg/ml) were measured by Luminex on day 76. **c** (%) of antigen induced specific CD4^+^ T cell responses from mice splenocytes were measured by intracellular cytokine staining (ICS) assay on day 76. Data is shown as the mean with standard deviation (SD). **d** (%) of antigen specific CD8^+^ T cell responses from mice splenocytes were measured by intracellular cytokine staining (ICS) assay on day 76. Data shows the median from 5 animals/group. Data is shown as the mean with SD.

T cell-mediated immunity has been described to play a critical role in the prevention of HZ through maintenance of latent VZV in a subclinical state in sensory ganglia^27^. Thus, we designed studies in mice to perform detailed evaluation of CD4^+^ T cell and CD8^+^ T cell responses elicited by the three lead VZV gE modRNA vaccine candidates, in addition to antibody responses. To this end, C57BL/6 mice (Jackson) were primed with a full subcutaneous human dose of Varivax® (Merck), a pediatric VZV live-attenuated vaccine (LAV), to resemble a VZV infection and natural chickenpox-induced immunity to VZV antigens. Five weeks after initial VZV LAV exposure (Day 35), mice were administered 1 µg of each modRNA/LNP or an equivalent dose of Shingrix or saline control. For reference, a human dose of Shingrix is 50 µg. Antigens were delivered intramuscularly in a prime and boost regimen 4 weeks apart (Fig. 6a). A similar strategy was previously followed by Fochesato et al. to assess the immunogenicity of a gE recombinant protein containing the AS01 adjuvant^28^.

In this Varivax®-primed mouse model experiment, the top three VZV modRNA selected candidates were tested as frozen formulations as well as a lyophilized version of gE_WT Co2. Antibody responses were evaluated via a Luminex assay to quantify the VZV gE-binding IgG levels in serum, while gE-specific T-cell responses were assessed by intracellular cytokine staining (ICS) assay to quantify IFN-γ, IL-2 and/or TNF-α-producing CD4^+^ and CD8^+^ T cells.

In mice that were previously primed with Varivax®, antibody responses measured 2 weeks after the boost (day 76) were comparable between the VZV modRNA candidates and the Shingrix® control at the 1 µg dose level tested. Notably, the gE_WT Co2 lyophilized version also performed similar to its frozen counterpart (Fig. 6b).

Analysis of the gE-specific T-cell responses at day 76 revealed that all the modRNA candidates induced strong CD4^+^ and CD8^+^ T cell responses (Figs. 6c, d). The percentage of IFN-γ-expressing CD4^+^ T cells elicited by the gE_WT Co2 modRNA vaccine candidate and its lyophilized version were substantially higher than Shingrix®, while the magnitude of CD4^+^ T cells induced by the membrane-bound mutant gE_ms5 Co1 and the secreted version gE_ms6 Co2 were comparable to Shingrix®.

Additionally, we evaluated the ability of the VZV modRNA vaccine candidates to induce polyfunctional T cell responses, defined as the ability to produce multiple cytokines such as IFN-γ, IL-2 and TNF-α. The stacked graphs depict the proportion of gE-specific activated CD4^+^ and CD8^+^ T cells that express either one, two or three cytokines (Figs. 6c, d). Results highlight that all three proposed VZV modRNA vaccine candidates: gE_WT Co2 (both frozen and lyophilized versions), gE_ms5 Co1 and gE_ms6 Co2 induced high proportions of CD4^+^ T cells expressing three cytokines that are comparable to Shingrix®. A strong CD8^+^ T cell response was induced by all VZV modRNA tested, while it was almost undetectable in Shingrix®-vaccinated mice (Fig. 6d). Overall, these findings highlight the ability of our proposed VZV modRNA vaccine candidates to elicit a robust T cell and antibody-mediated immune responses in a Varivax®-primed mouse model.

### Lyophilized modRNA constructs are stable over time and maintain immunogenic properties

**Fig. 7:**
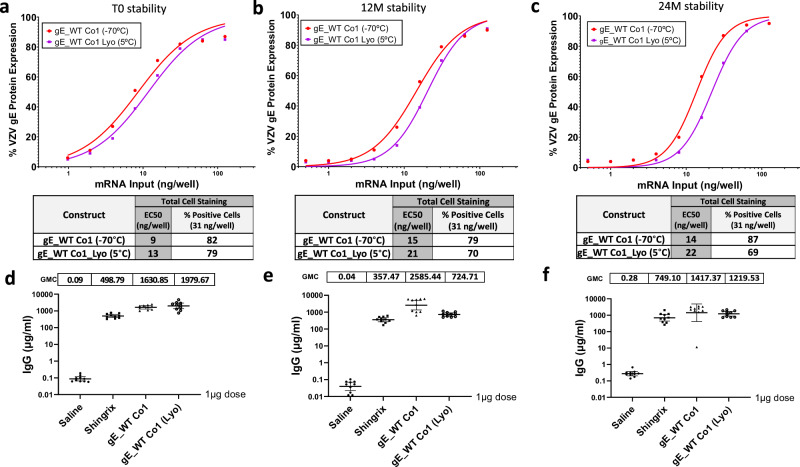
Stability and immunogenic profile of lyophilized mRNA material over time. In vitro expression (IVE) of frozen and lyophilized material for gE WT Co1 candidate was measured by flow cytometry in HEK 293 T cells at initial timepoints (**a**) as well as after 12 months (**b**) and 24 months (**c**). Whole gE expression levels were detected using a specific antibody against VZV gE. EC50 values (ng/well) were calculated from each curve and are shown in a table together with the (%) positive cells at 31 ng dose for all three conditions tested. Blood from mice previously immunized with 1 µg dose of Shingrix® or VZV gE WT Co1 (frozen or lyophilized) mRNA material preserved for 0 months (**d**), 12 months (**e**) or 24 months (**f**) was collected via submandibular route and gE-specific IgG antibodies (µg/ml) were measured by Luminex on day 76.

A lyophilized modRNA vaccine presentation that could be kept at refrigerated temperatures and reconstituted upon administration could help improve shelf-life stability of a modRNA vaccine. To that end, gE_WT Co1, was initially selected and subjected to a two-year stability assessment. A frozen formulation stored at −70 °C and a lyophilized formulation kept at 5 °C demonstrated similar EC50 values in an IVE assay at time 0 (Fig. 7a), 12 months (Fig. 7b) and 24 months (Fig. 7c). While overall trends show that the lyophilized version had slightly higher EC50 values compared to the frozen version, the expression was maintained up to 24 months with minimal loss in potency, thus indicating that the lyophilization process had minimal impact on the potency of the vaccine after two years at 5 °C.

In addition, these two formulations were evaluated at the same timepoints for immunogenicity levels after a prime-boost regimen and compared to the Shingrix® control. For this experiment, naïve BALB/c mice that were not previously exposed to VZV (or primed with Varivax®) were used. VZV gE IgG binding levels were similar for the frozen and lyophilized products, and both were markedly higher when compared to 1 µg Shingrix® at the initial timepoint tested (0 months) (Fig. 7d) as well as after 12 and 24 months when compared to Shingrix® (Figs. 7e, f). These overall results highlight the stable profile of a lyophilized modRNA vaccine candidate.

## Discussion

In this work, we have developed several VZV modRNA vaccine candidates targeting the glycoprotein gE, one of the most abundant proteins on the surface of the virion. VZV gE has been previously shown to be highly immunogenic and sufficient to provide high levels of protection against HZ when administered as an adjuvanted protein subunit vaccine^10^. This vaccine was licensed in 2017 under the name of Shingrix® (GlaxoSmithKline, Rockville, MD, US). While efficacy levels are over 90% across all groups tested^10,11^, moderate side effects are frequently reported in patients ≥50 years after vaccination^10^, which can be perceived as a burden by patients.

The mRNA technology offers several advantages when compared to other modalities such as adjuvanted subunit vaccines: mRNAs are easy to manufacture, can be produced faster compared to traditional vaccines, and are very versatile since modifications can be easily introduced^29^. mRNAs are delivered enclosed in a cationic LNP formulation to facilitate cellular uptake and endosomal escape and represent a novel and efficient platform to express different vaccine antigens. Additionally, Covid-19 mRNA vaccines have been shown to be very efficacious^30,31^ with an acceptable safety and tolerability profile. Vaccine reactogenicity depends on many factors including age, platform, formulation, antigen design, dose level and previous exposure to the antigen among others. It is not known yet if a VZV mRNA vaccine will have a similar tolerability profile to Covid-19 mRNA vaccines. Thus, our aim was to generate a more tolerable vaccine that could offer similar protection levels to Shingrix®.

A total of 19 different modRNA vaccine candidates were screened in vitro in Vero cells to evaluate gE protein expression levels, antigen subcellular localization and two different codon optimizations based on different GC content. Based on these parameters, 3 modRNA constructs were ultimately selected for in vivo assessment: gE WT Co2, ms5 Co1 and ms6 Co2. It is well known that protein localization can ultimately impact the induction of a strong immune response. Based on our confocal microscopy studies, the VZV gE WT version (gE_WT Co2) accumulated mostly intracellularly in the TGN^22^, which might be a suboptimal compartment for inducing proper humoral and cellular responses. The second selected vaccine candidate, ms5 Co1, is an endocytic recycling mutant of 581 amino acids that lacks a conserved YXXL domain that is key for endocytosis and efficient internalization of the protein^23^, which translated into gE being mainly expressed at the cell membrane. The last selected construct, ms6 Co2, is a secreted version that completely lacks the TM domain. As expected, this construct is mostly found in the supernatant of transfected cells. ms6 Co2 secreted slightly better than ms3 Co2, which contains part of the TM domain. We demonstrated that by removing the additional TM sequence, secretion of the gE antigen could be enhanced. Additionally, while confocal microscopy studies performed in Vero cells with DS showed more gE protein localized at the cell membrane for gE_ms5 co1 compared to gE_WT Co2, IVE performed in HEK-293T with LNP formulated material showed comparable levels between these two constructs when looking at gE cell surface staining levels. These results could be due to less efficient RNA delivery of LNPs compared to transfected DS material and also to the fact that different cell lines may differ in their ability to endocytose the gE protein and accumulate it in the Golgi, as this process was more effective and visible in Vero cells compared to HEK-293T cells. All three modRNA constructs were formulated into LNP drug product (DP) and were used to immunize mice that had been vaccinated 5 weeks prior with a full dose of Varivax®, a pediatric VZV live-attenuated vaccine, to create pre-existing immunity against the virus. Mice were primed with 1 µg of each selected VZV modRNA candidates or an equivalent dose of Shingrix® and boosted with an additional dose 4 weeks later. Vaccination with all three modRNA vaccine candidates were able to induce high IgG levels that were comparable to the Shingrix® control at two weeks post boost. As previously mentioned, Shingrix® consists of a protein subunit vaccine that is combined with a strong adjuvant (AS01B). Subunit vaccines have a favorable safety profile but low to moderate immunogenicity levels. To overcome this, they need to be combined with a strong adjuvant. However, adjuvants are responsible for many adverse effects both systemically and locally at the site of administration^10^. Notably, we have shown that immunizing mice with 1 µg of our lead modRNA candidates can elicit similar antibody responses to Shingrix®. This suggests that our investigational VZV modRNA vaccine could potentially have a better tolerability profile since no additional immunostimulatory compounds need to be added to the current formulation. Further clinical evaluations are needed to confirm this.

Regarding cell-mediated immune (CMI) responses, all the modRNA candidates were able to induce comparable or higher antigen-specific CD4^+^ T cell responses compared to Shingrix®. Induction of CMI has been previously reported for other vaccines expressing surface glycoproteins like gE^32,33^. The membrane bound mutant (gE_ms5 Co1) and the secreted version (gE_ms6 Co2) exhibited a comparable percentage of positive CD4^+^ T cells while numbers were slightly higher for the gE WT version (gE_WT Co2). All three modRNA vaccine candidates were also able to induce low but consistent CD8^+^ T cell responses, while these responses were not detectable upon Shingrix® administration. This mouse model recapitulates the findings of Shingrix® vaccination in humans, where CD8^+^ T cell responses were not elicited^34^. Delivery of LNP-formulated mRNA is perceived by cells as a virus-like stimuli but without the risk of potential viral replication and integration into the genome^35^. Intracellular presentation and activation of innate immune pathways can lead to efficient CD8^+^ T cell responses through MHC class I antigen presentation. However, and in line with our results, such CD8^+^ T cell responses are not expected with a subunit protein vaccine like Shingrix® since antigens are presented mostly through antigen presenting cells and MHC class II molecules. This is in part attributed to the adjuvant system used, where AS01_B_ has been shown to induce high gE-specific CD4^+^ T cell responses when compared to an aluminum-salt adjuvant^36^. However, there is also recent evidence of both CD4^+^ and CD8^+^ T cells playing an important role in controlling viral replication in ganglia during active HZ^37^. While vaccination with Shingrix has shown that CD8^+^ T cell responses are not necessary to confer protection, having both T cell responses activated upon modRNA vaccination could potentially translate into better protection when administered into humans, thus providing an additional layer of control that could reinforce vaccine efficacy.

In the preclinical work published by Cao et al., the authors described the vaccine efficacy in mice of a modRNA VZV gE construct that contains carboxy-terminal mutations. In that study, a C-terminal mutant along with a full length gE and an extracellular domain construct similar to the antigen used in Shingrix® were tested^38^. CMI responses were assessed for the three modRNA constructs using C57BL/6 mice. Results showed the modRNA vaccine candidate containing C-terminal mutations elicited the highest increase in both CD4^+^ and CD8^+^ T cell memory responses, especially when looking at CD44^+^/CD62L^+^ central memory T cell indicators. Another study recently published by Wang et al., that evaluated three VZV gE mRNA vaccine candidates, found that the gE-M-P construct containing a CT double mutation to target gE to the TGN or the plasma membrane, induced significantly higher CMI responses compared to Shingrix®. Such CMI responses have been demonstrated to be key for the prevention of HZ^39^. Our results are also consistent with the data recently published by Bhattacharya et al., where the authors assessed the antibody and T cell responses of an mRNA vaccine in mice based on three different VZV gE variants: a full length, a soluble and a truncated version of the protein by altering one of the TGN localization motifs^21^. Similar to our findings, these authors observed high antibody as well as potent antigen-specific CD4^+^ T cell responses. However, gE-specific CD8^+^ T cell responses were very low. The difference in CD8^+^ T cell responses observed between our study and Bhattacharya et al. could potentially be attributed to the different LNP formulations used in each study. Bhattacharya et al. used as a cationic lipid NOF or SM102 for their mRNA LNPs, while our drug product contains ALC-0315 which is known to induce very robust antigen-specific CD8^+^ T cell responses following intramuscular immunization^40,41^. These data also highlight that not all mRNA vaccines are equivalent. Immune responses must be considered holistically, and the induction of CD8^+^ T cell responses from our formulation in addition to antibodies and CD4^+^ T cell responses, could provide more robust protection.

Interestingly, regarding VZV CMI responses upon vaccination, different outcomes have been documented by different groups depending on the animal model tested. For instance, Monslow et al. did not observe CD8^+^ T cell responses in NHP, particularly rhesus macaques, upon modRNA administration^17^. These animals are non-permissive to VZV replication although they can mount cellular and humoral immune responses^42,43^. Despite the absence of antigen-specific CD8^+^ T cell responses in NHP, the same authors mentioned that they observed CD8^+^ T cell responses in response to modRNA in other animal models based on unpublished data^17^. Besides NHP, other animal models have been evaluated to better understand the host response to VZV infection. These include guinea pigs, rats, and mice. In our mouse study, antigen- specific CD8^+^ T cell responses were consistently observed for all three modRNA constructs. However, like in NHPs, there is a lack of viral replication in mice which limits the use of this animal model to investigate VZV-associated pathogenesis and immunity. In summary, due to the strict host specificity of VZV infection, to date there is still no animal model that could perfectly recapitulate the key clinical and virological features of VZV infection and reactivation. Therefore, further efforts should be focused towards developing better animal models that could mimic all aspects of VZV infection in humans. Moreover, recent findings have also shown that T cell responses are strongly dependent on LNP composition and therefore should be considered as another component that can contribute to the overall immune response^44^.

In addition to the goal of generating a modRNA VZV vaccine with high efficacy and a favorable reactogenicity profile, another aspect we focused on was the improvement of storage conditions. The Pfizer-BioNTech COVID-19 modRNA vaccine, Comirnaty®, previously relied on an ultra-frozen presentation and is available now in a refrigerated liquid format which is stable for up to 8 months from the manufacturing date. To further improve storage conditions, we explored the possibility of having a lyophilized modRNA vaccine presentation that could be kept stable at refrigerated temperatures for a longer duration. To address this, one of our modRNA constructs (gE_WT Co1) was selected to undergo a two-year stability assessment. To date, we have observed similar protein expression levels and immunogenicity between the lyophilized version that was kept at 5 °C and the -80 °C frozen formulation control when tested in vitro and in vivo after two years. Comparable high IgG titers between the frozen and lyophilized formulations were measured and both formulations produced higher titers than the Shingrix® control at the 0, 12 and 24 month timepoints.

In summary, we have generated and evaluated different modRNA vaccine candidates based on the glycoprotein gE for the prevention of HZ. Preclinical results obtained using in vivo models have shown that robust cellular and humoral responses are elicited upon vaccination. Some of these investigational VZV modRNA candidates, including a lyophilized presentation, are currently being tested in a Phase I/II clinical study (NCT05703607).

## Methods

### Design and Cloning of VZV-gE antigen candidates

Genes of interest (GOI) based on the VZV-gE antigen with different mutations, truncations and codon optimizations (Co1 and Co2) were designed based on the Ellen Iowa Strain (NCBI Ref: AH009994.2). Two wild-type (WT) constructs and a total of additional 17 different designs (ms3 to ms12) were made based on two different codon optimizations as well as modifications in the cytoplasmic tail (CT) and the transmembrane (TM) domain and are summarized in Fig. 1. GOIs were gene synthetized using BioXP (Telesis Bio) or PCR amplified (Invitrogen Platinum SuperFi-II 12368010). For cloning purposes, Gibson assembly (NEB Builder HiFi DNA Assembly NC0825276) or standard restriction digestion with SpeI (NEB, R3133L) and XhoI (R0146L) followed by ligation (Promega, PR-M1794) into our proprietary linearized vector were performed^45,46^.

Vector backbone, UTRs and poly A length were the same as previously described for Pfizer-BioNTech Covid-19 modRNA vaccine BNT162b2^47^. DH10-beta competent *E. coli* cells (NEB C3019H) were used for bacterial transformation of plasmids. Sequence was confirmed through direct colony sequencing (Templiphi amplification kit, Cytiva 45-002-266) followed by in-house Sanger Sequencing using BigDye Terminator v3.1 Cycle Sequencing Kit (Applied Biosystems 43-374-55). All primers for cloning and sequencing purposes were purchased from IDT (Integrated DNA Technologies).

### In-vitro transcription (IVT)

DNA plasmids where GOI were cloned under T7 promoter were first linearized with BspQI and further purified using phenol-chloroform extraction to generate the templates for in-vitro transcription. modRNAs were generated using CleanCap AG (Trilink, N7113-1). At the end of the reaction, turbo DNase (2 U/µl) (ThermoFisher, AM2238) was added to remove DNA template from the IVT reaction. modRNA material generated for in vitro screening purposes was purified using Invitrogen Dynabeads MyOne Carboxylic Acid (ThermoFisher, 65011). RNA material produced for animal studies was purified by tangential flow filtration (TFF) and 0.2 µm filtered. RNA integrity was determined by capillary electrophoresis (Agilent, Fragment Analyzer). RNA capping levels were determined by LC-UV. Concentration, pH, and residual endotoxin were also determined using standard laboratory procedures.

### Indirect immunofluorescence assay (IFA)

Vero cells (ATCC, CClL-81) were used to test drug substance (DS) or LNP-formulated material (DP). DP was directly delivered into cells by diluting the LNPs in Dulbecco’s phosphate-buffered saline (DPBS) without Calcium and Magnesium (Gibco). DS was delivered into cells using lipofectamine MessengerMax (Invitrogen, LMRNA015) and following manufacturer’s guidelines. After 16 h incubation period, transfection media was removed. Cells were then washed 3 times with PBS, fixed with 4% paraformaldehyde (PFA) (Invitrogen, 28908), and washed twice with 3% BSA (Millipore Sigma, 126626). Cells were stained with human monoclonal antibody against VZV gE (303-1A8) that was generated in house as previously described^48^. Antibody was diluted in a solution containing 0.1% saponin (Alfa Aesar) in 10% normal goat serum (Life Technologies, 50197Z). Antibody was incubated for 1 hour at 37 °C, followed by 3 washes with PBS. Alexa Fluor 488 secondary antibody (Invitrogen, A11013) was prepared at a 1:500 dilution along with a 1:140,000 dilution of HCS CellMask Orange Stain (Invitrogen, H32713) in the same solution previously used to incubate the primary antibody and incubated for 45 min at 37 °C. For nuclear cell staining, a 1:10,000 dilution of DAPI (Thermo Scientific, 62248) was incubated for 15 min at RT. After 3 washes with PBS, 96 well plates (Perkin Elmer, 6055508) were sealed and scanned with Opera Phenix Plus High Content Screening System (Perkin Elmer).

### Western blot (WB) analysis

HeLa cells (ATCC, CCL-2) were transfected with 2.5 µg of modRNA per well using a 6-well plate format. 24 h post-transfection, cell lysates and supernatants were processed separately to obtain protein extracts for WB analysis. Supernatants were collected and centrifuged at 1000 x *g* for 5 min to remove cellular debris. Cell lysates were collected by adding 250 µL RIPA buffer (Thermo Scientific, 89900) containing protease inhibitors (Thermo Scientific, A32965) and further combined with fresh OptiMEM to keep a consistent volume between the cell lysate and supernatant samples. Samples were aliquoted and stored at -80 °C.

For WB analysis, 10 µL of each sample was combined with NuPAGE 4X LDS Sample Buffer and NuPAGE 10X Sample Reducing Agent to a final concentration of 1X. The samples were denatured at 95 °C for 5 min and loaded on a NuPAGE 4–12% Bis-Tris gel (Invitrogen, NP0323BOX) along with 2 µL PageRuler Prestained Protein Ladder (Thermo Scientific, PI26616). The gel was run at 200 V (constant) for 60 min in 1X MOPS running buffer (Invitrogen, NP0001), and then transferred to a PVDF membrane (Invitrogen, IB24002) using the iBlot2 device (Invitrogen, IB21001) with the P0 transfer protocol. The blot was probed using anti-VZV gE protein mouse monoclonal antibody (Abcam, AB272686) at a 1:1,000 dilution followed by Goat anti-Mouse IgG (H + L) alkaline phosphatase conjugated secondary antibody (Invitrogen, G21060) at a 1:1,000 dilution. The membrane was probed with alkaline phosphatase chemiluminescent substrate (Novex, WP20002) for 5 min and then imaged using an iBright FL1000 imager.

### Imaging and quantification analysis

Vero cells were transfected with DS and imaged on an Ultraview Spinning Disk confocal microscope using a 63X oil objective and a Hamamatsu EMCCD camera. Ten images were taken per construct for a whole image quantification analysis and/or 2–3 regions of interest (Golgi organelle) per image for the cells only expressing the VZV gE protein specifically. Quantification analysis to measure the Pearsons’ correlation coefficient was carried out using the Costes et al., algorithm^49^ for background subtraction embedded within the Volocity software. Data was analyzed using the GraphPad Prism. Significance was assessed by one-way ANOVA which was available within the GraphPad Prism functions.

### LNP formulation method

Purified VZV mRNA was formulated into lipid nanoparticles (LNP) as previously described^46^. LNPs were formed by using impingement jet mixing of lipid mixture in ethanol and nucleic acid in low-pH buffer. The LNPs were then buffer exchanged to an aqueous buffer matrix via diafiltration and followed by final compounding and terminal filtration. Lipid mixture in ethanol comprised (ALC-0315(4-hydroxybutyl)azanediyl)bis(hexane-6,1-diyl)bis(2-hexyldecanoate)), a PEGylated lipid, 2-[(polyethylene glycol)-2000]-N,N-ditetradecylacetamide and two structural lipids (1,2-distearoyl-sn-glycero-3-phosphocholine (DSPC)and cholesterol). All formulations were stored at –70 °C or 5 °C conditions. Selected formulations were lyophilized (Lyostar 3 Freeze Dryer, SP Scientific, Warminster, PA) and stored at 5 °C. All LNP formulations were tested for particle size by Dynamic Light Scattering, mRNA encapsulation and concentration by RiboGreen Assay, RNA integrity by capillary electrophoresis (Agilent Fragment Analyzer) and in vitro expression (IVE) by cell-based flow cytometry.

### In vitro expression (IVE) assay

In vitro Expression (IVE) of VZV gE modRNA LNP formulated material was assessed by flow cytometry. HEK 293 T cells (ATCC) were seeded into 12-well culture plates (ThermoFisher) at 200,000 viable cells/well in 1 mL of growth media. Growth media consists of DMEM containing high glucose, GlutaMax and sodium pyruvate (ThermoFisher), and supplemented with 10% Fetal Bovine Serum Certified One Shot (Gibco). Once confluency was reached after 16–24 h, cells were transfected with the corresponding modRNA DPs serially diluted in Dulbecco’s phosphate-buffered saline (DPBS) without Calcium and Magnesium (Gibco). A total of 11-point 2-fold titrations in duplicate wells in a final volume of 100 µl/well were tested. Plates were further centrifuged at 550 RCF for 5 minutes at room temperature (RT). Transfected plates were kept in a 37 °C and 5% CO2 incubator for 16–24 h. Then, culture media was aspirated, and cells were detached with Accutase™ Cell Detachment Solution (Corning) by incubation at 37 °C with 5% CO2 for 10 min. Cells were resuspended in DPBS and harvested to U-bottom 96-Well Polystyrene Round Bottom Microwell Plates by pelleting using a temperature-controlled centrifuge at 550 RCF for 5 min at 25 °C. Pelleting was repeated between all further wash/treatment steps. Harvested cells were stained with LIVE/DEAD™ Fixable Aqua Dead Cell 405 Stain Kit (Invitrogen) following manufacturer’s guidelines. For detection of whole cell gE protein expression levels (intracellular and surface) fixation/permeabilization solution and permeabilization wash solution was used. In the case of surface cell protein expression, Cytofix™ Fixation Buffer (BD Biosciences) and Pharmingen Stain Buffer (BSA) were used. Cells were incubated in fixation solution for 20 minutes at 5 °C and further washed and pelleted. Cells were then resuspended in 50 µl of Mouse Anti-VZV gE primary antibody solution (Abcam, ab272686) [125 ng/ml] in wash buffer and incubated for 60 min at 5 °C followed by two washes to prepare for addition of 50 µl of secondary antibody solution [500 ng/ml] (Alexa Fluor® 488 AffiniPure Donkey Anti-Mouse IgG (H + L), (Jackson ImmunoResearch Laboratories) previously diluted in wash buffer and incubated further for 30 min at 5 °C. Cells were washed, pelleted, and supernatants were aspirated and finally resuspended in 200 µl of wash buffer. Cells were then acquired using LSRII or ORD Fortessa Flow Cytometry instruments and data were analyzed by FlowJo Software (BD Life Sciences). Data were processed by log-10 transformation of the x-axis and sigmoidal 4-PL non-linear curve fitting. Top and bottom of the curves were constrained to 100% and 0%, respectively. EC50 values were calculated from each curve using built-in functions of GraphPad Prism software.

### Animal immunizations, blood collection and tissue harvest

For the LAV primed mouse model, female C57BL/6 mice (10–15/group) were initially immunized subcutaneously on Day 0 with a full human dose of Varivax® (1350 pfu) in a total volume of 0.5 mL and subsequently vaccinated intramuscularly (IM) with VZV WT_gE CO2 (frozen or lyophilized) or Shingrix® control at a dose level of 1.0 µg in 50 µL at day 35 and day 63, respectively. Blood was collected via the submandibular route on day 76 and processed for serum for antibody testing by Luminex. No anesthesia was required for submandibular bleeds and animals were manipulated while awake. Approximately 150 µL of whole blood was collected dropwise directly into microtainer tubes containing serum separators. For terminal blood draws, exsanguination was performed via cardiac puncture. For this procedure, animals were induced to a surgical plane of anesthesia via inhalation of aerosolized isoflurane (2–4%). After terminal blood collection, animals were cervically dislocated as a secondary method of euthanasia. At all collection time points, blood tubes remained at room temperature for at least 30 minutes prior to centrifuging at 10,000 RPM for 3 min. Samples were stored at −80 °C until testing.

Spleens were also harvested on Day 76 (C57BL/6 mice) to analyze CMI responses. The harvested tissues were placed on a 100 µm size cell strainer immersed in 7 mL of cRPMI (10% FBS/RPMI) per mouse per well of 6-well plate. The plates were maintained on ice during transit and before processing for single cell suspension of cells. Spleens were subjected to RBC lysis and passage through cell strainer to remove RBCs and clumps.

Regarding the immunogenicity mouse model for stability, female BALB/c mice (10/group) were vaccinated with VZV gE_CO2 (frozen or lyophilized) or Shingrix® control at a dose level of 1 µg at day 0 and day 28 respectively. Blood was collected via the submandibular route on day 42 and processed for serum for antibody testing by Luminex. Approximately 150 µL of whole blood was collected dropwise directly into microtainer tubes containing serum separators. For terminal blood draws, the entire available blood volume was collected via cardiac puncture. At all collection time points, blood tubes remained at room temperature for at least 30 min prior to centrifuging at 10,000 RPM for 3 minutes. Samples were stored at -80 °C until testing.

### Ethics statement for in vivo studies

Mouse immunogenicity studies were performed at Pfizer, Inc. (Pearl River, NY, US), which is accredited by the Association for Assessment and Accreditation of Laboratory Animal Care (AAALAC). All procedures were performed in accordance with local regulations and established guidelines and were reviewed and approved by an Institutional Animal Care and Use Committee (IACUC).

### gE-binding IgG luminex assay

A soluble strep-tagged VZV gE recombinant protein similar to VZV gE_ms3 (gE ZgE223 Strep tag) produced in house was coupled to Single-Plex microspheres (Luminex). VZV gE coated microspheres were diluted in blocking buffer to 50,000 beads/mL and incubated for 1–2 h at room temperature with shaking immediately prior to addition to serially diluted mouse sera, controls, or the reference standard: a mouse monoclonal antibody against VZV gE protein (Abcam, ab272686). Serum IgG bound to VZV gE was detected with a R-phycoerythrin-labeled goat anti-mouse IgG secondary antibody (F(ab’)2 Fragment Specific; Jackson ImmunoResearch Laboratories, 115-116-072). The magnitude of the fluorescent signal measured by a Luminex FLEXMAP 3D reader is directly proportional to the amount of bound anti-VZV gE IgG. The data were analyzed using a custom SAS application, which uses a log/log linear regression model of the standard curve to interpolate antigen-specific antibody concentrations (µg/mL) from median fluorescent intensity.

### gE-specific T-cell responses

Vaccine-induced T-cell responses to VZV gE in mouse splenocytes were measured by intracellular cytokine staining (ICS) assay. Ex-vivo stimulation of splenocytes in the presence of gE peptide pool activates production of various cytokines such as IFN-γ in the antigen-specific CD4^+^ and CD8^+^ T cells that were analyzed by flow cytometry in ICS assay.

Single cell suspensions of splenocytes (2 × 10^6^ cells/well) were cultured ex vivo in cRPMI with media-DMSO (unstimulated) or VZV gE peptide pool (JPT, Berling Germany, 15aa, 11aa overlap) for 5 h at 37 °C in the presence of anti-CD107a APC antibody and protein transport inhibitors, GolgiPlug and GolgiStop. Following stimulation, splenocytes were stained for viability and then incubated with fluorescently conjugated antibodies in flow cytometry (FC) buffer (2% FBS/PBS) to stain for surface proteins CD19, CD3, CD4, CD8, CD44 (25 ± 5 min at room temperature, RT). Cells were fixed and permeabilized before staining for intracellular proteins IFN-γ, TNF-α, IL-2 and CD40L/CD154 (25 ± 5 min at 18–25 °C). After staining, the cells were washed and resuspended in FC buffer. Cells were acquired on LSR Fortessa and data analyzed by FlowJo (10.7.1). Results are background (media-DMSO) subtracted and shown as percentage of cytokine-expressing CD4^+^ T cells and CD8^+^ T cells, respectively.

## Supplementary information

Supplementary Information

## Data Availability

All data generated or analyzed during this study are included in the published article and its supplementary information file. Primary data sets may be made available upon reasonable request and with the permission of Pfizer Inc.
