# Supplementary material for: A highly stable lyophilized mRNA vaccine for Herpes Zoster provides potent cellular and humoral responses
Source: NPJ Vaccines. 2025 Mar 14;10:49. doi: 10.1038/s41541-025-01093-1 (PMC11909110; doi:10.1038/s41541-025-01093-1)
Supplement: Supplementary file 1 — Supplementary Information [file 41541_2025_1093_MOESM1_ESM.pdf]

Supplementary Figure 1

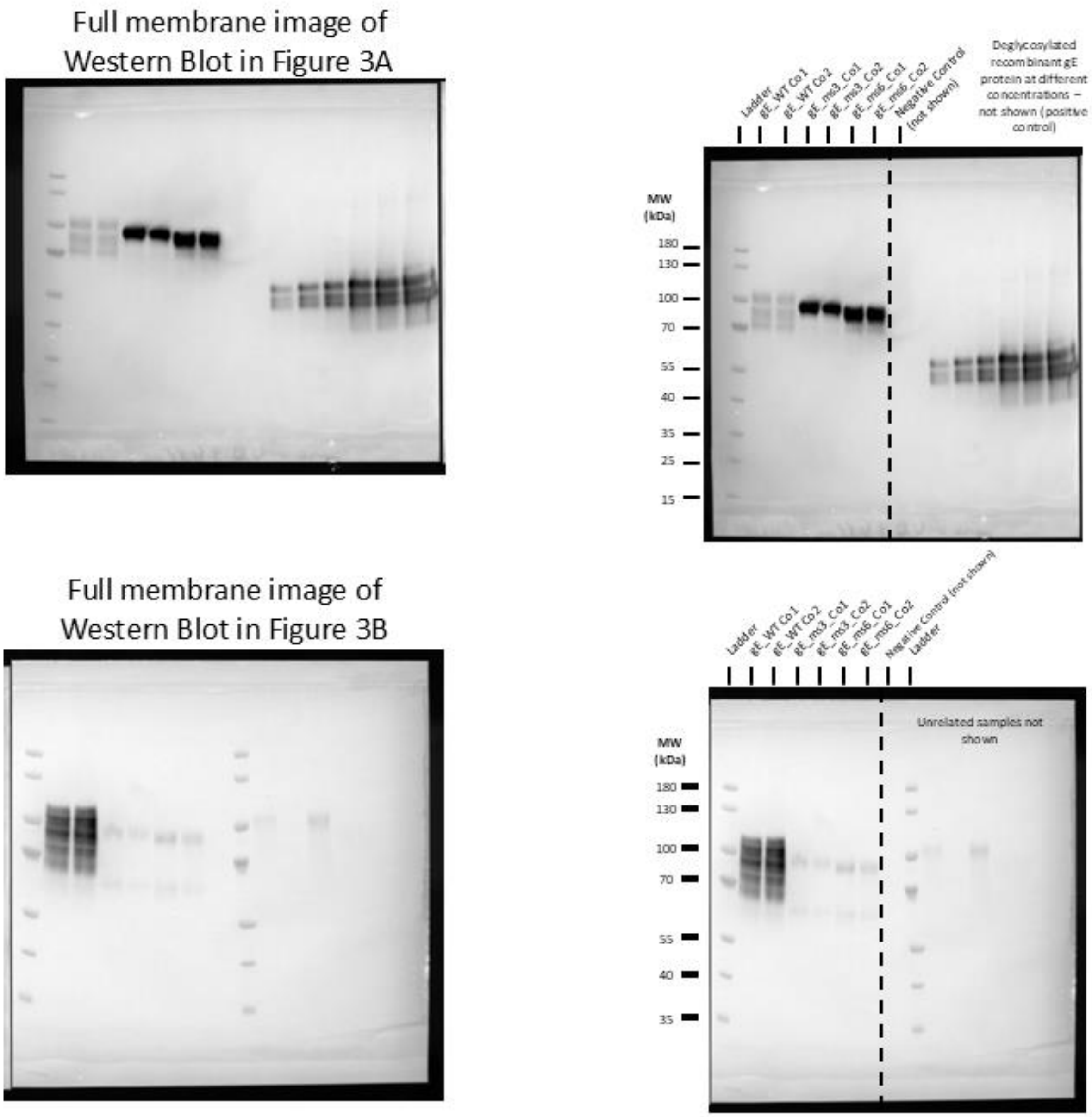

Supplementary Figure 1. mRNA gE constructs expressing secreted versions of gE are highly expressed in cell supernatants. Unprocessed Western blots from Figure 3. Cell supernatants (A) and cell extracts (B) after transfecting HeLa cells with 2.5μg of RNA with the indicated gE-expressing mRNA constructs (gE WT, gE_ms3 and gE_ms6) that were synthetized with two different codon optimizations (Co1 and Co2). Samples were collected and analyzed 24h after transfection. For the blot from Figure 3B, gE WT constructs for each codon optimization serve as positive controls. Cells were transfected with mRNA encoding GFP as a negative control.
